# Representation of female nurses in journalistic texts in Bahia from 1936 to 1956*

**DOI:** 10.1590/1980-220X-REEUSP-2024-0199en

**Published:** 2025-06-06

**Authors:** Victor Porfirio Ferreira Almeida Santos, Monalisa Viana Sant’Anna, Maria Itayra Padilha, Fernando Rocha Porto, Luciana Barizon Luchesi, Gilberto Tadeu Reis da Silva

**Affiliations:** 1 Universidade Federal da Bahia, Escola de Enfermagem, Salvador, BA, Brazil.; 2 Universidade Federal Santa Catarina, Programa de Pós-Graduação em Enfermagem, Florianópolis, SC, Brazil.; 3 Universidade Federal do Estado do Rio de Janeiro, Escola de Enfermagem Alfredo Pinto, Rio de Janeiro, RJ, Brazil.; 4 Universidade de São Paulo, Escola de Enfermagem de Ribeirão Preto, Ribeirão Preto, SP, Brazil.

**Keywords:** History of Nursing, Nursing, Mass Media, Social Identification, Public Health

## Abstract

**Objective:**

To analyze the representations of female nurses, published in the written press in Bahia, as a strategy of visibility for the society in Bahia, from 1936 to 1956.

**Method:**

Historical study with a serial approach, in the dimension of Cultural History and in the field of the history of nursing and female nurses, which used as data sources the reports published in the newspaper from Bahia called A TARDE, from 1936 to 1956, and discussions on themes of History of Nursing, in Rio de Janeiro and Bahia, articulated with the adherence literature.

**Results:**

A total of 542 publications were found with three representations of nurses in the journalistic press: public health nurse, military nurse, and religious nurse.

**Conclusion:**

The representation constructed and disseminated in the newspaper from Bahia was historically used for social, economic, and political purposes over time, through the various interested sectors, and began to compose a professional identity, which, to this day, interferes with the recognition and visibility of the profession.

## INTRODUCTION

The journalistic text currently presents itself as a possibility for writing history and new perspectives on the use of sources in research, within the scope of New History. This is a complex field, as the journalistic text seeks to legitimize a world view, to give credibility to something. As a consequence, they influence the political and economic fields, promoting changes or maintaining the status of different segments^(1)^. For Nursing, the media, which includes journalistic texts, is a space for fighting for visibility and professional recognition. At the same time, it sometimes creates a space for misinformation about the role of the profession and for maintaining stereotypes.

Several representations are presented by the media through sets of explanations, images, and concepts that underpin collective ideas about professional identity. However, the identity of the profession is related to the continuous construction, which involves nursing and its interaction with the world and the struggle for recognition of professional identity versus the social representation of this identity^(2)^.

This representation has consequences in the social imaginary of stereotypes about female activity, by identifying them as religious, submissive, obedient, docile, fragile in their critical and reflective positions towards the job market, which ended up having the effect of a certain vision of precarious work in professional practice^(3,4)^. It is worth remembering that this representation lasted for a long time and only in more recent times has it been progressively modified. The COVID-19 pandemic, for example, contributed to substantially changing the previous image, to a more political, proactive, empowered one, although still with remnants of angels, heroines, among other stereotypes^(5)^, which, due to their anachronism and distance from reality, create barriers to professional appreciation and recognition^(4)^. The dissemination of images and ideas about nursing in the media over time helps to identify advances and setbacks, as well as the establishment of professional visibility strategies.

In this context, aiming at contributing to this critical construction, the objective of the present study is to investigate and analyze how nursing was portrayed in the print media in the first half of the 20th century. To this end, we contextualize that, until the beginning of the 1920s, ten Nursing Schools were already established in Brazil, on the Rio-São Paulo hub, and some of them already presented a certain distinction in their object representations. As examples, we can mention the Professional School of Nurses of the *Hospital Nacional de Alienados* (Rio de Janeiro, 1890), which would later become the Nursing School Alfredo Pinto of the Universidade Federal do Estado do Rio de Janeiro - UNIRIO (Rio de Janeiro - 1942), which had as its representation the student/nurse as an assistant to the doctor with the symbol of the cap as a personal head attribute; the Practical School of Nurses of the Brazilian Red Cross (Rio de Janeiro - 1916), whose representation was that of a kind and charitable nurse due to the veil on her uniform; and the School of Nurses of the National Department of Public Health, currently the Nursing School Anna Nery (Rio de Janeiro - 1923), where the nurse was seen as a social intervener, intermediary between the patient and the doctor^(6)^.

Other schools of the period were portrayed in journalistic texts or through documentary evidence, such as the Nursing Course at Hospital Samaritano, created in 1894, when it brought the Nightingale system to Brazil, but without the intention of implementing it. We can also mention the Nursing Course at the Maternity Hospital of São Paulo and the Nursing Course at the Hospital São Joaquim, both in 1908; the Nursing School of the Brazilian Red Cross, São Paulo Branch, in 1914 (all in the city of São Paulo); and, in 1918, the Nursing-Midwives Course at Pró-Matre and the Nursing Course at the Polyclinic of Botafogo, both in Rio de Janeiro^(7)^.

The Anglo-American Nightingale nursing model emerged in the early 1920s, with the implementation of the School of Nurses of the National Department of Public Health, in 1922^(8)^, and contributed to increasing the visibility of the profession in the media. The attributes expected to represent a female nurse were mentioned, especially by doctors, and included patience, selflessness and sensitivity, which were especially distinct from male nurses at the time^(6)^. On the other hand, Ethel Parsons led the cooperation project between the Brazilian and North American governments and transformed the development of Brazilian nursing with its own identity^(8)^.

The institutional representations of three of these schools – Professional School of Nurses, Practical School of Nurses of the Brazilian Red Cross and School of Nurses of the National Department of Public Health had their effects on drug advertising, in the period from 1920 to 1925, with 90 advertisements published in the Fon-Fon Magazine^(9)^. Furthermore, when the temporality was extended (1910–1931), the representations, which also referred to the signs of nurses in educational institutions, showed that they were, in addition to messengers of the identities of educational institutions, representative references of advertising pieces^(10)^.

The understanding of these historical representations in the state of Bahia, specifically, refers to the period from 1923 to 1925, when health campaigns became frequent and subsidized investments in public health. In the 1930s, the Federal Health Departments encouraged exchanges with the Bahia Public Health Department in teaching in health area^(11)^. This occurred due to the precariousness of public healthcare services provided by hospitals in Bahia^(12)^.

Based on this scenario, the then dean Edgard Santos, of the Universidade Federal da Bahia (*UFBA*), proposed the creation of a hospital complex, which would require the work of registered nurses. Therefore, to meet this need, the Director of the Nursing School at Universidade de São Paulo (*USP*), nurse Edith de Magalhães Fraenkel, was invited to implement the project of the Nursing School of Bahia (*EEB*)^(11)^.

It should be noted that the *EEB* was created by Decree Law No. 8.779, of January 22, 1946, and incorporated into the *UFBA* when it assumed the objective of training nurses to provide assistance to the new university hospitals that were in the inauguration phase and, with this, promote health education^(12)^.

Thus, in the wake of these events and recognizing the historical and social trajectory of the construction of the image of the nurse in society, the present study aims to analyze the representations of nurses, through the written press in Bahia, as a strategy of visibility for the society in Bahia, from 1936 to 1956.

## METHOD

### Design of Study

This is a historical study in the serial approach, in the dimension of Cultural History and in the domain of the history of nursing and women, which means, respectively, the typology of the sequenced source (newspaper), when it is possible to investigate part of the reality experienced^(13)^, and, in this case, the representation of the nurse.

### Local

The time frame from 1936 to 1956 covers the 20-year period of the process of coordination between the Federal Health Departments and the Public Health Secretariat (Bahia) in teaching activities in the health field. The trajectory shows that the image of the nurse was used by social, economic, and political interests over time through interested sectors. In this time frame, they had as context the Vargas Era, World War II, and the creation of the Nursing School of the Universidade Federal da Bahia, thus influencing the formation of their identity and professional visibility.

Therefore, the geographical delimitation is limited to the state of Bahia and has as its priority documentary source the journalistic text, specifically the series of the newspaper “A Tarde”, due to its circulation in Bahia, with editions from October 1912 to the present. The aforementioned newspaper was founded by Ernesto Simões Filho (1886–1957), a law graduate and founding member of the Bahia Academy of Letters^(14)^.

### Selection Criteria

The inclusion criterion used was the presence of the term nurse exclusively in the feminine in Portugues, and the exclusion criterion was the term in the masculine, in addition to news about nursing outside the state of Bahia.

### Data Collection

The search in the “A Tarde” newspapers took place on the website of the Pedro Calmon Foundation, located in Salvador (BA), from July 2018 to January 2019. The findings were organized in a *Microsoft Excel* spreadsheet composed of the following variables: date, title, and page.

### Data Analysis and Treatment

The process of triangulation of data sources was used as a method of analysis, allowing the synthesis and analysis of the content and its possible interrelations and distances from the themes^(15)^, as well as subsequent discussion on the themes of History of Nursing, in Rio de Janeiro and Bahia, articulated with the literature.

The analysis of the publications in the database was carried out using the ATLAS.ti software (*Qualitative Research and Solutions*) from the coding of journalistic excerpts from the newspaper “A Tarde”, with the terms female nurse(s) in each report. Through this procedure, the data triangulation technique was applied, based on specific criteria in light of the reported context and its codification for the narrative of qualitative data, ensuring credibility and reliability. Furthermore, there was also the elaboration of the descriptive statement, when it was produced by the characterized female nurse(s), giving rise to the categories^(16)^.

### Ethical Aspects

The investigation followed the standards defined in Resolutions No. 466/2012 and No. 510/2016 of the National Health Council, as well as in Law No. 12.527/2011 on copyright. There was no need to submit to the Ethics Committee, as it was a documentary research that used only public documents freely accessible to the population.

## RESULTS

In this race to represent nurses, several adjectives were adopted with a focus on representing what this nurse meant in her singularity. Some of these adjectives were: Registered Nurse; Practicing Nurse; Volunteer and Professional Nurse; Social Nurse Visitor; Public Health Nurse; Catholic Nurse; Auxiliary Nurse; First Aid Nurse; and Samaritan Nurse^(17)^.

In the symbolic struggle to enunciate the representation of the nurse, such adjectives were published in the press for decades, until the enactment of Law No. 775/1949, which provided for nursing education in the country. This legal device established two denominations in nursing training: Nurse and Nursing Assistant distinguished by prerequisites, training time, and content taught.

**Figure 1:**
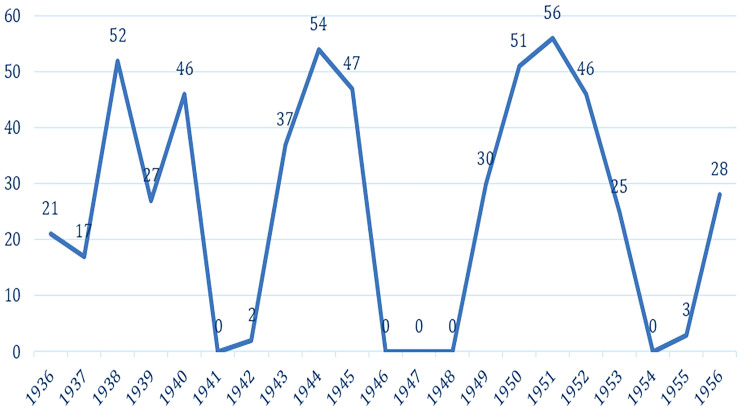
Movement of publications with the term nurse(s) in the newspaper A Tarde (1936-1956) – Salvador, BA, Brazil, 2019.

That said, 542 publications in the newspaper “A Tarde”, from 1936 to 1956, are detailed in Figure 1. Analysis of these publications pointed to three more compelling representations of nurses at the time: *public health nurse, military nurse, and religious nurse.* It is worth noting that, in the years 1941, 1946, 1947, 1948 and 1954, no mentions of the term nurse(s) were found.

In this context, the representations of nurses, through the serialized discourse of the newspaper “A Tarde”, culturally reveal one of the parts of the dominance of women’s history. Soon, it floated in three adjectives as a mental image about the feminine in Bahia.

In the research in the newspaper “A Tarde”, the excerpts analyzed with the occurrences of the term *public health nurse* were used in reference to graduates of the Nursing School of the National Department of Public Health/Nursing School Dona Anna Nery. The public health nurse in the country contributed to the deconstruction of the contextual determinants in relation to the work of the nurse in the country. The inclusion of DNSP registered nurses in society brought recognition and minimization of medical resistance, in the face of the assistance recommended in the prevention and promotion of health.

We then move on to the second representation found in this study, that of *military nursing.* At the end of the 1930s and 1940s, World War II (1939–1945) broke out. At the time, the Brazilian Red Cross – the central agency in Rio de Janeiro – promoted the Samaritan Nurses Course, with an emphasis on training war nursing, in accordance with its institutional principles, which can be summarized in the expression of acting in peace and war for the benefit of humanity^(18)^. From this perspective, news stories were published to attract candidates for training before they left for the war scene.

Therefore, the military training of nurses for the war front, going abroad and returning to Brazilian lands was the subject of studies^(19-20)^. The articles published in the newspaper “A Tarde” presented the criteria for admission to the Brazilian Expeditionary Force: certificate in nursing or of Samaritan, marital status (single or widowed), age between 20 and 40 years, moral suitability, and physical fitness. Furthermore, they linked this decision to feelings of patriotism and heroism, associated with the image of Anna Nery. However, focusing on the representation of the military nurse, in the scenario of Bahia there are speeches of appeals to “call” women.

The articles also reported on the role of women in the war and brought with them the representation of the female hero. This referred to interpretative signs in two aspects: 1) to minimize consciousness, when the symbol is determined for that which is part of the known; and 2) to determine human attitudes towards itself, resulting in the rupture or maintenance of power^(21)^. In this case, both were conveyed in association with military women, that is, moral examples, fighters, strong spirits, performers of magnificent and brave work.

Regarding the third representation found, about the *religious nurse* in the newspaper “A Tarde”, the articles were associated with the attributes of charity, vocational gift, and benevolence. In this sense, they related the profession to the practice of care by nuns (Sisters of Charity), still in the 19th century, before the beginning of the process of professionalization of nursing in Brazil.

We emphasize that the act of charity carried out by the nuns was, in fact, strategically praised by hospital managers. This perception was evident in some fragments of the newspaper “A Tarde”, which mentioned the impossibility of receiving an unhealthy work bonus, as a labor right, because they were exercising charity. Therefore, the representation of a religious nurse was covered with feelings of doctrinal vows in favor of the divine.

Other articles in the newspaper “A Tarde” referred to the personification of the representation of the religious nurse devoid of vanity, which reiterated the image of angels in white, assistants of God on earth and humanitarians^(22)^. In this way, the association strengthened the sacralization and further obscured who, in fact, was a professional nurse.

## DISCUSSION

The 1920s were marked by the Health Reform, led by Carlos Chagas, and the implementation of Modern Nursing. At the time, there was strong investment in the written and illustrated press about the Nursing School of the National Department of Public Health, currently the Nursing School Anna Nery, and also about the Practical Nursing School of the Brazilian Red Cross – central agency – Rio de Janeiro^(6)^. In addition to these, other educational institutions in favor of professionalization were also in the dispute for the enunciation of the mental representation of the nurse.

Such representations (public health, military and religious nurse), identified in this study, have, in fact, their roots in the past. As mentioned, the School of Nursing in Bahia was created in 1946, on the initiative of UFBA^(11)^. However, to better understand this process, we need to go back to the 1920s, when public health actions in the state of Bahia were reconfigured. Consequently, the focus of policies in the health field was directed towards health with an emphasis on health education and the control of prevalent infectious diseases, in different geographic areas of society of Bahia, in the period from 1925 to 1930, induced by the federal sphere. However, the 1930 Revolution led to the decline of that health reform due to the new adaptations of the government at the time^(23)^.

In the 1920s, the School of Nurses of the Sub-Secretariat of Health and Public Assistance (EESSAP) was included in the text of the Sanitary Code (1925) and suggested the implementation of an educational institution in the state of Bahia, considering the Sanitary Nursing Service. Such a service would then be centralized in commanding public health actions with a view to assisting field authorities and carrying out hygiene surveillance, as well as the control and prevention of infectious diseases and health education in private and educational spaces for basic training^(24)^.

It is important to mention that these requirements for student enrollment coincided with those required for students who wished to attend the School of Nursing of the National Department of Public Health, in Rio de Janeiro. The public health nurse of the 1920s, in Rio de Janeiro, in fact, demarcated her space in Brazilian nursing and in the health field under the direction of North American nurses Clara Louise Kieninger, Lorraine Genevi and Bertha Pullen, under the leadership of Ethel Parsons, from 1923 to 1931. It was also a period of significant financial investment in the institutional functioning of the School by the Rockefeller Foundation^(8)^.

We know that, until 1929, Dr. JJ Fontenelle, in a document found in the Documentation Center of the Anna Nery Nursing School (EEAN - CD, mod. A, box 25, doc. 55, 1930), reported the presence of 93 public health nurses trained by the Nursing School of the National Department of Public Health. However, 63% of them were no longer in the profession, in the field of public health, and the predictions were not promising^(25)^. This is confirmed in another study, according to which 70% of graduates preferred less stressful services, such as private and/or hospital work, to the detriment of home visits^(26)^.

Thus, based on the premises outlined, we identified that female nurses were looking for other types of services, including those free from the problems of home visits and exposure to pathogenic agents, to take on other positions in health institutions. However, during Ethel Parsons’ management of Brazilian nursing, she built and gave visibility to the representation of nurses in public health by feeding (back) the collective imagination that women could be economically emancipated by pursuing this career^(8)^.

In parallel with the exodus of nurses from the public health field to other spaces, it is worth highlighting the end of the Rockefeller Foundation’s subsidy to the school, Parsons’ departure to Texas, when Decree No. 20.109/1931 was enacted, making Nursing School Anna Nery the official standard school for training nurses in Brazil. Such facts may lead some to interpret that the legal device was the national institutionalization of nursing.

The representation of nurses in public health, as advocated by Parsons, was promising for the emancipation of women, especially financially, when choosing a profession^(8)^. Such discourse, for the 1920s, in the midst of the women’s suffrage movement, was motivating and bold, since the arduous activity in the field was unknown, considering the physical wear and tear of home visits, exposure to pathogenic agents, and remuneration, resulting in an exodus to the hospital field^(25,26)^. In this regard, the discourse conveyed in the newspaper “A Tarde” about the representation of the public health nurse points to signs of sensitizing appeal.

The context of the 1920s and beyond served as a driving force for women to at least have the desire to be nurses and, as a result, become financially emancipated. The fact is that the discourse conveyed in the newspaper “A Tarde” somehow reached the female audience and may have influenced the creation of the *EEB* in the 1940s, which reinforced the arguments for its target^(11)^.

The representation of the military nurse was glorified by the idea of the heroine endorsed by Eurico Gaspar Dutra, Minister of War (1942–1945), in journalistic guidelines. These publications were covered in adjectives related to the feminine, such as love, selflessness, servile and disciplinary stance^(19)^.

The symbolic weight of the representation of the military nurse associated with the term heroine articulated with the memory of the trajectory of Anna Nery, native of Bahia, was, in fact, an emblematic appeal to Brazilian women and, especially, to women from Bahia. Therefore, we concluded that it was a matter of recording the participation of women in the pages of National History in the context of the war linked to the Brazilian Expeditionary Force, to strengthen this representation in the collective imagination.

Considering the presentation of the identity of nurses in the religious context, it is a historical construction of a practice that involved ideals of humility, love for others, submission, demarcated with the advent of Christianity, which taught these ideals to its followers, but also imposed them on care practices. This fact led to a strong connection between Christian ideals and nursing work^(27)^.

Thinking along these lines means understanding that the representation of the 19th century nurse, for example, was not so far removed from what was advocated in the 1910s and 1920s. The assertion shows us that, between 1930 and 1950, it remained in the journalistic discourse in the newspaper “A Tarde”. Nursing work carries within itself influences from the origins of health practices, associating women with concepts of religiosity, maternal spirit, or submission. This points to a certain haze in the representation of this professional, given the confusion with religious aspects, even in the face of regulations, so that this distortion prevailed in the collective imagination as a legacy of the sociocultural construction of millennia^(22,28)^.

In view of the above, we understand that, regardless of the representation of the nurse in the historical facts disclosed from the perspective of journalism (public health, military or religious nurse), this profession was marked in sociocultural minds as a feminine activity and by its social construction. This finding, reiterated in this study, serves as a mental trigger for a critical reflection on the constitution of this representation and its visibility with the aim of raising people’s awareness about the real social role of nurses in health, the relevance of their work, and the formation of professional identity.

Possible limitations include the use of only the term nurse(s), in the feminine in Portuguese, to search for other sources, and the selection of a single newspaper.

As contributions, this study intended to shed light on the representations of nurses in journalistic texts of Bahia in the first half of the 20th century, which contributes to other studies from a historical perspective to be carried out, to understand the identity movements of the profession. Furthermore, it may support new research on the representations of female nurses from Bahia and the Northeast region of the country.

## CONCLUSION

The representation of the nurse published in the print media in the newspaper “A Tarde” between 1936 and 1956 consisted of a triad: articulation with public health, military nurse, and religious imagery of the nurse. The representation constructed and disseminated in the newspaper from Bahia was historically used for social, economic, and political purposes over time, through the various interested sectors, and began to compose a professional identity, which, to this day, interferes with the recognition and visibility of the profession.

The historical period between 1936 and 1956 was notable for activities involving care and the actions of the people who carried out these practices. In this sense, nursing was consolidated as a necessary social activity present in war conflicts and in the context of community health practices, as observed in the present analysis of journalistic excerpts from the time.

Therefore, the present study allowed carrying out a critical reflection on the representation of nurses in the face of historical events reported by the print media in Bahia, which favors awareness of their social role in health, the relevance of their work, and the construction of professional identity.

Reaching this moment does not, however, mean its end. What we have is the tip of the iceberg on the representation of the nurse in another investigative layer beyond the Rio-São Paulo hub, with other investments by researchers in their states being needed to investigate the topic addressed.

## Data Availability

The full dataset supporting the findings of this study is available upon request to corresponding author Victor Porfirio Ferreira Almeida Santos.
