# Supplementary material for: Representation of female nurses in journalistic texts in Bahia from 1936 to 1956
Source: Rev Esc Enferm USP. 2025 Jun 6;59:e20240199. doi: 10.1590/1980-220X-REEUSP-2024-0199en (PMC12172669; doi:10.1590/1980-220X-REEUSP-2024-0199en)
Supplement: Translation

# Translation: Representação da enfermeira no texto jornalístico baiano de 1936 a 1956*

**DOI:** 10.1590/1980-220X-REEUSP-2024-0199pt

**Authors:** Victor Porfirio Ferreira Almeida Santos, Monalisa Viana Sant’Anna, Maria Itayra Padilha, Fernando Rocha Porto, Luciana Barizon Luchesi, Gilberto Tadeu Reis da Silva

**Affiliations:** 1 Universidade Federal da Bahia, Escola de Enfermagem, Salvador, BA, Brasil.; 2 Universidade Federal Santa Catarina, Programa de Pós-Graduação em Enfermagem, Florianópolis, SC, Brasil.; 3 Universidade Federal do Estado do Rio de Janeiro, Escola de Enfermagem Alfredo Pinto, Rio de Janeiro, RJ, Brasil.; 4 Universidade de São Paulo, Escola de Enfermagem de Ribeirão Preto, Ribeirão Preto, SP, Brasil.

**Keywords:** História da Enfermagem, Enfermagem, Meios de Comunicação de Massa, Identificação Social, Saúde Pública

## Abstract

**Objetivo:**

Analisar as representações das enfermeiras, divulgada na imprensa escrita na Bahia, como estratégia de visibilidade para a sociedade baiana, no período de 1936 a 1956.

**Método:**

Estudo histórico com abordagem serial, na dimensão da História Cultural e no domínio da história da enfermagem e das enfermeiras, que utilizou como fontes de dados as reportagens publicadas no Jornal baiano A TARDE, no período de 1936 a 1956, e discussões sobre temas de História da Enfermagem, no Rio de Janeiro e na Bahia, articuladas com a literatura de aderência.

**Resultados:**

Foram identificadas 542 publicações com três representações da enfermeira na imprensa jornalística: enfermeira em saúde pública, enfermeira militar e enfermeira religiosa.

**Conclusão:**

A representação construída e divulgada no jornal baiano foi historicamente utilizada para fins de interesses sociais, econômicos e políticos ao longo do tempo, por meio dos diversos setores interessados, e passou a compor uma identidade profissional, a qual, até os dias atuais, interfere no reconhecimento e visibilidade da profissão.

## INTRODUÇÃO

O texto jornalístico apresenta-se na atualidade como uma possibilidade de escrita da história e novos olhares sobre uso de fontes na pesquisa, no âmbito da História Nova. Trata-se de campo complexo, pois o texto jornalístico busca legitimar uma visão de mundo, dar credibilidade a algo. Como consequência, influenciam o campo político, econômico, promovem mudanças ou manutenção de status de diferentes segmentos^(1)^. Para a Enfermagem, a mídia, que inclui o texto jornalístico, é espaço de luta por visibilidade e reconhecimento profissional. Ao mesmo tempo, por vezes, constitui espaço de desinformação sobre o papel da profissão e de manutenção de estereótipos.

Diversas representações são apresentadas pela mídia por conjuntos de explicações, imagens e conceitos que alicerçam ideias coletivas sobre a identidade profissional. Entretanto, a identidade da profissão relaciona-se a contínua construção, que envolve a enfermagem e sua interação com o mundo e a luta por reconhecimento da identidade profissional versus a representação social dessa identidade^(2)^.

Tal representação tem consequências no imaginário social de estereótipos sobre a atividade feminina, por identificá-las como religiosas, submissas, obedientes, dóceis, frágeis em seus posicionamentos críticos e reflexivos para o mercado de trabalho, o que acabou tendo por efeito certa visão de trabalho precário no agir profissional^(3,4)^. Vale lembrar que esta representação perdurou por muito tempo e apenas em momentos mais recentes vem sendo progressivamente modificada. A pandemia de COVID-19, por exemplo, contribuiu para modificar substancialmente a imagem anterior, para outra mais política, proativa, empoderada, embora ainda com resquícios de anjos, heroínas, dentre outros estereótipos^(5)^, que geram, por seu anacronismo e distanciamento da realidade, barreiras para valorização e reconhecimento profissional^(4)^. A veiculação de imagens e ideias a respeito da enfermagem na mídia ao longo do tempo ajudam a perceber avanços e retrocessos, bem como o estabelecimento de estratégias de visibilidade profissional.

Nesse contexto, com o intuito de contribuir para essa construção crítica, o objetivo do presente estudo é investigar e analisar de que forma a enfermagem foi retratada na mídia impressa na primeira metade do século XX. Para tanto, contextualizamos que, até o início da década de 1920, dez Escolas de Enfermagem já estavam estabelecidas no Brasil, no eixo Rio-São Paulo, e algumas delas já apresentavam certa distinção nas suas representações objetais. Como exemplo, citamos a Escola Profissional de Enfermeiros e Enfermeiras do Hospital Nacional de Alienados (Rio de Janeiro, 1890), que futuramente seria a Escola de Enfermagem Alfredo Pinto da Universidade Federal do Estado do Rio de Janeiro - UNIRIO (Rio de Janeiro - 1942), que tinha por representação a estudante/enfermeira como auxiliar do médico com o signo do gorro como atributo pessoal de cabeça; Escola Prática de Enfermeiras da Cruz Vermelha Brasileira (Rio de Janeiro - 1916), cuja representação era a de enfermeira bondosa e caridosa pelo véu em seu uniforme; e Escola de Enfermeiras do Departamento Nacional de Saúde Pública, atual Escola de Enfermagem Anna Nery (Rio de Janeiro – 1923), como interventora social, intermediadora entre o doente e o médico^(6)^.

Outras Escolas do período foram retratadas no texto jornalístico ou por vestígios documentais, como o Curso de Enfermeiras do Hospital Samaritano, criado em 1894, quando trouxe para o Brasil o sistema nightingaleano, mas sem a intencionalidade de sua implantação. Podemos mencionar, ainda, o Curso de Enfermeiras na Maternidade de São Paulo e o Curso de Enfermeiras do Hospital São Joaquim, ambos em 1908; a Escola de Enfermagem da Cruz Vermelha Brasileira, Filial de São Paulo, em 1914 (todos na cidade de São Paulo); e, em 1918, o Curso de Enfermeiras-Parteiras na Pró-Matre e o Curso de Enfermeiras da Policlínica de Botafogo, ambos no Rio de Janeiro^(7)^.

O modelo de enfermagem nightingaleano anglo-americano surgiu no início da década de 1920, com a implantação da Escola de Enfermeiras do Departamento Nacional de Saúde Pública, em 1922^(8)^, e contribuiu para ampliação da visibilidade da profissão na mídia. Os atributos esperados para representação da enfermeira eram ditos, especialmente, pelos médicos, e incluíam paciência, abnegação e sensibilidade, especialmente distintas dos enfermeiros à época^(6)^. Por outro lado, Ethel Parsons liderou o projeto de cooperação entre os governos brasileiros e norte-americanos e transformou o desenvolvimento da enfermagem brasileira com identidade própria^(8)^.

As representações institucionais de três dessas escolas – Escola Profissional de Enfermeiros e Enfermeiras, Escola Prática de Enfermeiras da Cruz Vermelha Brasileira e Escola de Enfermeiras do Departamento Nacional de Saúde Pública tiveram seus efeitos nas publicidades de medicamentos, no período de 1920 a 1925, com 90 anúncios veiculados na Revista Fon-Fon^(9)^. Ademais, quando ampliada a temporalidade (1910–1931), as representações, que também remetiam aos signos das enfermeiras das instituições de ensino, evidenciaram que elas eram, para além de mensageiras das identidades das instituições de ensino, referências representativas das peças publicitárias^(10)^.

A compreensão dessas representações históricas no estado da Bahia, especificamente, remete ao período de 1923 a 1925, quando as campanhas sanitárias se tornaram frequentes e subsidiaram investimentos na saúde pública. Na década de 1930, as Delegacias de Saúde Federais favoreceram o intercâmbio com a Secretaria de Saúde Pública da Bahia com o ensino no campo da saúde^(11)^. Isso ocorreu em virtude da precariedade dos serviços assistenciais públicos oferecidos pelos hospitais baianos^(12)^.

Com base nesse cenário, o então reitor Edgard Santos, da Universidade Federal da Bahia (UFBA), propôs a criação de um complexo hospitalar, o qual demandaria a atuação de enfermeiras diplomadas. Dessa forma, para atender a essa necessidade, foi convidada a Diretora da Escola de Enfermagem da Universidade de São Paulo (USP), a enfermeira Edith de Magalhães Fraenkel, para implantar o projeto da Escola de Enfermagem da Bahia (EEB)^(11)^.

Cabe ressaltar que a EEB foi criada por meio do Decreto Lei n 8.779, de 22 de janeiro de 1946, e incorporada à UFBA ao assumir o objetivo de formar enfermeiras para prestar assistência aos novos hospitais universitários que estavam em fase de inauguração e, com isso, promover a educação sanitária^(12)^.

Assim, na esteira desses acontecimentos e reconhecendo a trajetória histórica e social de construção da imagem da enfermeira na sociedade, o presente estudo tem por objetivo analisar as representações das enfermeiras, por meio da imprensa escrita na Bahia, como estratégia de visibilidade para a sociedade baiana, no período de 1936 a 1956.

## MÉTODO

### Tipo do Estudo

Trata-se de um estudo histórico na abordagem serial, na dimensão da História Cultural e no domínio da história da enfermagem e das mulheres, o que significa, respectivamente, a tipologia da fonte sequenciada (jornal), quando nele é possível investigar parte da realidade vivida^(13)^, e, neste caso, a representação da enfermeira.

### Local

A delimitação temporal de 1936 a 1956 contempla o período de 20 anos do processo de articulações das Delegacias de Saúde Federais e da Secretaria de Saúde Pública (Bahia) em atividades de ensino no campo da saúde. A trajetória mostra que a imagem da enfermeira foi utilizada por interesses sociais, econômicos e políticos ao longo do tempo por meio dos setores interessados. Nesse recorte temporal tiveram como contexto a Era Vargas, II Guerra Mundial e a criação da Escola de Enfermagem da Universidade Federal da Bahia, acarretando assim, uma influência na formação de sua identidade e visibilidade profissional

Portanto, a delimitação geográfica atém-se ao estado da Bahia e tem como fonte documental prioritária o texto jornalístico, especificamente a série do Jornal “A Tarde”, em virtude de sua circulação na Bahia, com edição de outubro de 1912 até o presente. O referido jornal foi fundado por Ernesto Simões Filho (1886-1957), formado em Direito e membro fundador também da Academia de Letras da Bahia^(14)^.

### Critérios de Seleção

O critério de inclusão utilizado foi a presença do termo enfermeira exclusivamente no feminino, e de exclusão o termo no masculino, além de notícias da enfermagem fora do estado da Bahia.

### Coleta de Dados

A busca nos jornais “A Tarde” ocorreu no sítio eletrônico da Fundação Pedro Calmon, localizada em Salvador (BA), no período de julho de 2018 a janeiro de 2019. Os achados foram organizados em planilha do *Microsoft Excel* composta pelas seguintes variáveis: datação, título e página.

### Análise e Tratamento dos Dados

O processo de triangulação das fontes de dados deu-se como método de análise, permitindo a síntese e análise do conteúdo e suas possíveis interrelações e distanciamentos dos temas^(15)^, assim como posterior discussão nos temas de História da Enfermagem, no Rio de Janeiro e na Bahia, articulados com a literatura de aderência.

A análise das publicações do banco de dados ocorreu pelo uso do software ATLAS.ti (*Qualitative Research and Solutions*) a partir da codificação dos trechos jornalísticos do Jornal “A Tarde”, com os termos enfermeira(s) em cada reportagem. Mediante este procedimento foi aplicada a técnica de triangulação dos dados, a partir de critérios próprios à luz do contexto noticiado e sua codificação para a narrativa dos dados qualitativos, garantindo a credibilidade e confiabilidade. Ademais, houve ainda a elaboração do enunciado descritivo, quando foi produzido pela(s) enfermeira(s) adjetivadas, dando origem às categorias^(16)^.

### Aspectos Éticos

A investigação seguiu as normas definidas nas Resoluções nº 466/2012 e nº 510/2016 do Conselho Nacional de Saúde, bem como na Lei nº 12.527/2011 sobre os direitos autorais. Houve dispensa de submissão ao Comitê de Ética, por ser uma pesquisa documental que utilizou apenas documentos públicos de acesso gratuito à população.

## RESULTADOS

Nessa corrida em prol da representação da enfermeira, várias adjetivações foram adotadas com cerne na representação do que significava esta enfermeira na sua singularidade. Constituíram algumas dessas adjetivações: Enfermeira Diplomada; Enfermeira Praticante/Prática; Enfermeira Voluntária e Profissional; Enfermeira Visitadora Social; Enfermeira de Saúde Pública; Enfermeira Católica; Enfermeira auxiliar; Enfermeira Socorrista; e Enfermeira Samaritana^(17)^.

Na luta simbólica para enunciar a representação da enfermeira, tais adjetivações foram veiculadas na imprensa ao longo de décadas, até a promulgação da Lei n. 775/1949, que dispunha sobre o ensino de enfermagem no país. Este dispositivo legal instituiu duas denominações na formação da enfermagem: Enfermeira e Auxiliar de Enfermagem de distinção pelos pré-requisitos tempo de formação e conteúdo ministrado.

Isto posto, 542 publicações no Jornal “A Tarde”, no período de 1936 a 1956 são detalhadas na Figura 1. A análise dessas publicações apontou para três representações mais contundentes das enfermeiras da época: *enfermeira em saúde pública, enfermeira militar e enfermeira religiosa.* Cabe destacar que, nos anos de 1941, 1946, 1947, 1948 e 1954 não foram encontradas menções ao termo enfermeira(s).

**Figura 1:** Movimento das publicações com o termo enfermeira(s) no Jornal A Tarde (1936-1956) – Salvador, BA, Brasil, 2019.

Nesse contexto, as representações das enfermeiras, por meio do discurso serializado do Jornal “A Tarde”, revelam culturalmente uma das parcelas do domínio da história das mulheres. Logo, ela flutuou em três adjetivações como imagem mental sobre o feminino no espaço baiano.

Na pesquisa no jornal “A Tarde”, os trechos analisados com as ocorrências do termo *enfermeira de saúde pública* foram usados em referência às egressas da Escola de Enfermagem do Departamento Nacional de Saúde Pública/Escola de Enfermeiras Dona Anna Nery. A enfermeira de saúde pública no país contribuiu para a desconstrução dos determinantes de contexto em relação ao trabalho da enfermeira no país. A inserção de enfermeira diplomada do DNSP na sociedade trouxe reconhecimento e minimização das resistências médicas, diante da assistência preconizada na prevenção e promoção da saúde.

Partimos, então, para a segunda representação encontrada neste estudo, a da *enfermagem militar.* No final da passagem da década de 1930 para 1940, eclodiu a II Guerra Mundial (1939–1945). Na época, a Cruz Vermelha Brasileira – órgão central, no Rio de Janeiro – promoveu o Curso de Enfermeiras Samaritanas, com ênfase no treinamento da enfermagem de guerra, conforme seus princípios institucionais, os quais podem ser sintetizados na expressão de atuação na paz e guerra em prol da humanidade^(18)^. Nessa perspectiva, matérias jornalísticas foram veiculadas para atrair candidatas ao treinamento, antes de partirem para o cenário bélico.

Assim sendo, o treinamento militar de enfermeiras para o front de guerra, ida para o exterior e retorno a terras brasileiras foi objeto de estudos^(19–20)^. As matérias veiculadas no Jornal “A Tarde” apresentavam os critérios para admissão na Força Expedicionária Brasileira: diploma de enfermeira ou samaritana, estado civil solteira ou viúva, idade entre 20 e 40 anos, idoneidade moral e aptidão física. Além disso, vinculavam essa decisão a sentimentos de patriotismo e heroísmo, associados com a imagem de Anna Nery. Contudo, atendo-nos à representação da enfermeira militar, no cenário baiano evidenciam discursos de apelos de “convocatória” às mulheres.

As matérias relatavam ainda a atuação das mulheres na guerra e traziam consigo a representação da mulher heroína. Isto remetia aos signos interpretativos em dois aspectos: 1) para minimizar as consciências, quando o símbolo é determinado para aquilo que faz parte do conhecido; e 2) para determinar as atitudes humanas com relação a ele mesmo, resultando na ruptura ou manutenção do poder^(21)^. Neste caso, ambos foram veiculados em associação à mulher militar, ou seja, exemplos morais, combatentes, fortalezas de espírito, executoras de trabalho magnífico e valentes.

No que se refere à terceira representação encontrada, acerca da *enfermeira religiosa* no Jornal “A Tarde”, as matérias foram associadas aos atributos de caridade, dom vocacional e benevolência. Nesse sentido, relacionavam a profissão à prática dos cuidados das religiosas (Irmãs da Caridade), ainda do século XIX, antes do início do processo de profissionalização da enfermagem no Brasil.

Ressaltamos que o ato de caridade exercido pelas religiosas era, inclusive, estrategicamente enaltecido pelos gestores dos hospitais. Essa percepção ficou evidente em alguns fragmentos do Jornal “A Tarde”, os quais mencionaram a impossibilidade de receberem gratificação de insalubridade, como direito trabalhista, por estarem no exercício da caridade. Logo, a representação de enfermeira religiosa era revestida de sentimentos de votos doutrinários em prol do divino.

Outras matérias do Jornal “A Tarde” referiam-se à personificação da representação da enfermeira religiosa desprovida de vaidade, o que reiterou a imagem de anjos de branco, adjuvantes de Deus na terra e humanitárias^(22)^. Dessa forma, a associação fortalecia a sacralização e brumava ainda mais quem, de fato, era a profissional enfermeira.

## DISCUSSÃO

A década de 1920 foi marcada pela Reforma Sanitária, liderada por Carlos Chagas, e pela implantação da Enfermagem Moderna. Na época, era forte o investimento na imprensa escrita e ilustrada acerca da Escola de Enfermeiras do Departamento Nacional de Saúde Pública, atual Escola de Enfermagem Anna Nery, e, também, sobre a Escola Prática de Enfermeira da Cruz Vermelha Brasileira – órgão central – Rio de Janeiro^(6)^. Além dessas, outras instituições de ensino em prol da profissionalização também estavam na disputa pela enunciação da representação mental da enfermeira.

Tais representações (enfermeira de saúde pública, militar e religiosa), identificadas neste estudo, têm, de fato, suas raízes no passado. Como dito, a Escola de Enfermagem na Bahia foi criada em 1946, por iniciativa da UFBA^(11)^, mas, para melhor entendimento desse processo, precisamos retroagir aos anos de 1920, quando as ações de saúde pública no estado da Bahia foram reconfiguradas. Assim, o foco das políticas no campo da saúde dirigiu-se à saúde com ênfase na educação sanitária e no controle das doenças infectocontagiosas prevalentes, em diferentes espaços geográficos da sociedade baiana, no período de 1925 a 1930, induzido pela esfera federal. Contudo, a Revolução de 1930 levou ao declínio aquela reforma sanitária para as novas adequações do governo à época^(23)^.

Nos anos de 1920, a Escola de Enfermeiras da Subsecretaria de Saúde e Assistência Pública (EESSAP) constava no texto do Código Sanitário (1925) e sugeria a implantação de uma instituição de ensino no estado da Bahia, considerando o Serviço de Enfermeiras Sanitárias. Tal serviço estaria, então, centralizado no comando das ações na saúde pública com vistas a auxiliar as autoridades do campo e executar a vigilância higiênica, bem como o controle e prevenção de doenças infectocontagiosas e a educação sanitária nos espaços privados e educacionais de formação básica^(24)^.

Importante mencionar que esses requisitos para matrícula de estudantes confluíram com aqueles exigidos para as estudantes que desejavam cursar a Escola de Enfermagem do Departamento Nacional de Saúde Pública, no Rio de Janeiro. A enfermeira de saúde pública da década de 1920, no Rio de Janeiro, de fato, demarcou seu espaço na enfermagem brasileira e no campo da saúde sob a direção das enfermeiras norte-americanas Clara Louise Kieninger, Lorraine Genevi e Bertha Pullen, sob a liderança de Ethel Parsons, no período de 1923 a 1931. Também foi um período de investimentos financeiros de monta para o funcionamento institucional da Escola pela Fundação Rockfeller^(8)^.

Sabemos que, até 1929, Dr. J. J. Fontenelle, em documento encontrado no Centro de Documentação da Escola de Enfermagem Anna Nery (EEAN - CD, mód.A, cx. 25, doc. 55, 1930), relatou a presença de 93 enfermeiras de saúde pública formadas pela Escola de Enfermagem do Departamento Nacional de Saúde Pública. Contudo, 63% delas não se encontravam mais na profissão, no campo da saúde pública, e as previsões não eram promissoras^(25)^. Isto é ratificado em outro estudo, segundo o qual 70% das diplomadas preferiam serviços menos desgastantes, tais como atividade particular e/ou hospitalares, em detrimento das visitas domiciliares^(26)^.

Isso posto, partindo das premissas apontadas, identificamos que as enfermeiras estavam em busca de outras modalidades de serviços, inclusive, livres das intempéries nas visitações domiciliares e das exposições a agentes patogênicos, para assumir outras posições nas instituições de saúde. Contudo, durante a permanência de Ethel Parsons na liderança da enfermagem brasileira, ela construiu e deu visibilidade à representação da enfermeira em saúde pública ao (retro) alimentar o imaginário coletivo de que a mulher poderia ser economicamente emancipada ao seguir essa carreira^(8)^.

Paralelamente ao êxodo das enfermeiras do campo da saúde pública para outros espaços, há de se ressaltar o final do subvencionamento da Fundação Rockefeller à escola, a partida de Parsons para o Texas, quando ocorreu a promulgação do Decreto nº 20.109/1931, tornando a Escola de Enfermeiras Anna Nery a escola oficial padrão para a formação de enfermeiras no Brasil. Tais fatos podem levar alguns à interpretação de que o dispositivo legal tenha sido a institucionalização nacional da enfermagem.

A representação da enfermeira em saúde pública, como preconizava Parsons, era promissora em prol da emancipação da mulher, especialmente a financeira, quando pela escolha profissional^(8)^. Tal discurso, para a década de 1920, em pleno movimento pelo sufrágio feminino, era motivador e ousado, desde que se desconhecesse a árdua atividade no campo, considerando o desgaste físico das visitas domiciliares, exposições aos agentes patogênicos e a remuneração, tendo por efeito o êxodo para o campo hospitalar^(25,26)^. Nesse sentido, o discurso veiculado no Jornal “A Tarde” sobre a representação da enfermeira de saúde pública aponta para indícios de apelo sensibilizadores.

O contexto da década de 1920 e seguinte servia de mola propulsora para as mulheres terem, ao menos, o desejo de serem enfermeiras e, por efeito, emancipadas financeiramente. Fato é que o discurso veiculado no Jornal “A Tarde” de alguma forma atingia o público feminino e pode ter influenciado a criação da Escola de Enfermagem da Bahia (EEB) na década de 1940, o que reforçava os argumentos para o seu objetivo^(11)^.

A representação de enfermeira militar era glorificada pela ideia de heroína endossada por Eurico Gaspar Dutra, ministro da Guerra (1942–1945), nas pautas jornalísticas. Essas publicações eram revestidas de adjetivações relacionadas ao feminino, tais como amor, abnegação, postura servil e disciplinar^(19)^.

O peso simbólico da representação da enfermeira militar associada ao termo heroína articulada à memória da trajetória da baiana Anna Nery era, de fato, um apelo emblemático às brasileiras e, especialmente, às baianas. Logo, depreendemos que se tratava de registrar a participação da mulher nas páginas da História Nacional no contexto da guerra vinculada à Força Expedicionária Brasileira, de maneira a fortalecer essa representação no imaginário coletivo.

Considerando a apresentação da identidade das enfermeiras no âmbito religioso, trata-se de construção histórica de uma prática que envolvia ideais de humildade, amor ao próximo, submissão, demarcados com o advento do cristianismo, que ensinava esses ideais para seus fiéis, mas também os impôs às práticas do cuidado. Esse fato provocou forte conexão dos ideais cristãos aos trabalhos de enfermagem^(27)^.

Pensar nessa linha é entender que a representação da enfermeira do século XIX, por exemplo, não estava tão distante do que se preconizava nas décadas de 1910 e 1920. A assertiva nos mostra que, entre 1930 e 1950, ela permanecia no discurso jornalístico no Jornal “A Tarde”. O trabalho da enfermagem carrega em si influências das origens das práticas de saúde associando a mulher a conceitos de religiosidade, espírito maternal ou submissão. Isso aponta para certa névoa na representação desta profissional, dada a confusão com os aspectos religiosos, mesmo diante das regulamentações, de modo que essa distorção prevaleceu no imaginário coletivo como uma herança da construção sociocultural de milênios^(22,28)^.

Diante do exposto, entendemos que, independentemente da representação da enfermeira nos fatos históricos divulgados sob a ótica do jornalismo (enfermeira de saúde pública, militar ou religiosa), tal profissão estava marcada nas mentes socioculturais como atividade feminina e pela sua construção social. Tal constatação, reiterada neste estudo, serve de gatilho mental para uma reflexão crítica sobre a constituição dessa representação e sua visibilidade no intuito de conscientizar as pessoas sobre o real papel social da enfermeira na saúde, a relevância do seu trabalho e a formação da identidade profissional.

Entende-se, como possíveis limitações, o uso apenas do termo enfermeira(s), no feminino, para a busca de outras fontes, e a escolha de um único jornal.

Como contribuições, este estudo pretendeu lançar luzes sobre as representações de enfermeiras no texto jornalístico baiano na primeira metade do século XX, o que contribui para outros estudos na perspectiva histórica a serem realizados, no sentido de compreender os movimentos identitários da profissão. Além disso, poderá subsidiar novas pesquisas acerca das representações de enfermeiras baianas e da região Nordeste do país.

## CONCLUSÃO

A representação da enfermeira divulgada na mídia impressa no Jornal “A Tarde” no período de 1936 a 1956 era constituída por uma tríade: articulação com a saúde pública, enfermeira militar e imagética religiosa da enfermeira. Essa representação construída e divulgada no jornal baiano foi historicamente utilizada para fins de interesses sociais, econômicos e políticos ao longo do tempo, por meio dos diversos setores interessados, e passou a compor uma identidade profissional, a qual, até os dias atuais, interfere no reconhecimento e visibilidade da profissão, ou seja, na forma como é vista e reconhecida pela sociedade.

O período histórico entre os anos de 1936 e 1956 destacou-se por atividades que contemplam o cuidado e pela atuação das pessoas que executavam essas práticas. Nesse sentido, a enfermagem consolidou-se como atividade social necessária e presente nos conflitos bélicos e no contexto das práticas de saúde à comunidade, tal como observado na presente análise dos trechos jornalísticos da época.

Assim, o presente estudo possibilitou realizar uma reflexão crítica sobre a representação da enfermeira diante dos acontecimentos históricos divulgados pela mídia impressa baiana, o que favorece a conscientização sobre seu papel social na saúde, a relevância do seu trabalho e a formação da identidade profissional.

Chegar a este momento não significa, contudo, seu término. O que temos é a ponta de *iceberg* sobre a representação da enfermeira em outra camada investigativa para além do eixo Rio-São Paulo, cabendo outros investimentos de pesquisadores em seus estados perscrutarem sobre a temática abordada.

## Data Availability

Todo o conjunto de dados que dá suporte aos resultados deste estudo está disponível mediante solicitação ao autor correspondente Victor Porfirio Ferreira Almeida Santos.

# Translation: Representación de la enfermera en los textos periodísticos bahianos de 1936 a 1956*

**DOI:** 10.1590/1980-220X-REEUSP-2024-0199es

**Authors:** Victor Porfirio Ferreira Almeida Santos, Monalisa Viana Sant’Anna, Maria Itayra Padilha, Fernando Rocha Porto, Luciana Barizon Luchesi, Gilberto Tadeu Reis da Silva

**Affiliations:** 1 Universidade Federal da Bahia, Escola de Enfermagem, Salvador, BA, Brasil.; 2 Universidade Federal Santa Catarina, Programa de Pós-Graduação em Enfermagem, Florianópolis, SC, Brasil.; 3 Universidade Federal do Estado do Rio de Janeiro, Escola de Enfermagem Alfredo Pinto, Rio de Janeiro, RJ, Brasil.; 4 Universidade de São Paulo, Escola de Enfermagem de Ribeirão Preto, Ribeirão Preto, SP, Brasil.

**Keywords:** Historia de la Enfermería, Enfermería, Medios de Comunicación de Masas, Identificación Social, Salud Pública

## Abstract

**Objetivo:**

Analizar las representaciones de las enfermeras, publicadas en la prensa escrita de Bahía, como estrategia de visibilidad para la sociedad de la Bahia, en el período de 1936 a 1956.

**Método:**

Estudio histórico con enfoque serial, en la dimensión de la Historia Cultural y en el campo de la historia de la enfermería y de las enfermeras, que utilizó como fuentes de datos los reportajes publicados en el periódico de Bahia A TARDE, de 1936 a 1956, y discusiones sobre temas de Historia de la Enfermería, en Río de Janeiro y Bahía, articulados con la literatura de adhesión.

**Resultados:**

Se identificaron 542 publicaciones con tres representaciones de enfermeras en la prensa periodística: enfermera de salud pública, enfermera militar y enfermera religiosa.

**Conclusión:**

La representación construida y difundida en el periódico de Bahia fue históricamente utilizada por intereses sociales, económicos y políticos a lo largo del tiempo, a través de los diversos sectores interesados, y pasó a componer una identidad profesional, que, hasta los días de hoy, interfiere en el reconocimiento y visibilidad de la profesión.

## INTRODUCCIÓN

El texto periodístico se presenta actualmente como una posibilidad de escritura de la historia y de nuevas perspectivas sobre el uso de las fuentes en la investigación, en el ámbito de la Nueva Historia. Es un campo complejo, ya que el texto periodístico busca legitimar una visión del mundo, dar credibilidad a algo. Como resultado, influye en las esferas política y económica y promueve cambios o el mantenimiento del estatus de diferentes segmentos^(1)^. Para la enfermería, los medios de comunicación, incluido el texto periodístico, son un espacio de lucha por la visibilidad y el reconocimiento profesional. Al mismo tiempo, a veces es un espacio de desinformación sobre el papel de la profesión y de mantenimiento de estereotipos.

Los medios de comunicación presentan diversas representaciones a través de conjuntos de explicaciones, imágenes y conceptos que sustentan las ideas colectivas sobre la identidad profesional. Sin embargo, la identidad de la profesión está relacionada con una construcción continua, que implica a la enfermería y su interacción con el mundo y la lucha por el reconocimiento de la identidad profesional frente a la representación social de esta identidad^(2)^.

Esta representación tiene consecuencias en el imaginario social de estereotipos sobre la actividad femenina, identificándolas como religiosas, sumisas, obedientes, dóciles y frágiles en sus posturas críticas y reflexivas ante el mercado laboral, lo que ha tenido como efecto la creación de una cierta visión de precariedad laboral en la actividad profesional^(3,4)^. Conviene recordar que esta representación ha perdurado durante mucho tiempo y sólo en épocas más recientes se ha ido modificando progresivamente. La pandemia del COVID-19, por ejemplo, ha contribuido a cambiar sustancialmente la imagen anterior por otra más política, proactiva y empoderada, aunque todavía tiene restos de ángeles, heroínas y otros estereotipos^(5)^ que, por su anacronismo y distancia de la realidad, crean barreras para el reconocimiento y la valoración profesional^(4)^. La difusión de imágenes e ideas sobre la enfermería en los medios de comunicación a lo largo del tiempo nos ayuda a comprender los avances y retrocesos, así como el establecimiento de estrategias de visibilidad profesional.

En este contexto, con el fin de contribuir a esta construcción crítica, el objetivo de este estudio es investigar y analizar cómo la enfermería fue retratada en la prensa escrita en la primera mitad del siglo XX. Para ello, contextualizamos el hecho de que, a principios de la década de 1920, ya se habían establecido diez escuelas de enfermería en Brasil, en el eje Río-São Paulo, y algunas de ellas ya mostraban cierta distinción en sus representaciones objetuales. Por ejemplo, la Escuela Profesional de Enfermeras del Hospital Nacional de Alienados (Río de Janeiro, 1890), que más tarde se convertiría en la Escuela de Enfermería Alfredo Pinto de la Universidad Federal del Estado de Río de Janeiro - UNIRIO (Río de Janeiro - 1942), representaba a la estudiante/enfermera como ayudante de médico con el signo del gorro como atributo personal de la cabeza; La Escuela Práctica de Enfermeras de la Cruz Roja Brasileña (Río de Janeiro - 1916), cuya representación era la de una enfermera bondadosa y caritativa a través del velo del uniforme; y la Escuela de Enfermeras del Departamento Nacional de Salud Pública, hoy Escuela de Enfermería Anna Nery (Río de Janeiro - 1923), como interventora social, intermediaria entre el paciente y el médico^(6)^.

Otras escuelas de la época fueron retratadas en el texto periodístico o por vestigios documentales, como el Curso de Enfermeras del Hospital Samaritano, creado en 1894, cuando el sistema “Nightingaleano” fue traído a Brasil, pero sin la intención de implantarlo. También podemos citar el Curso de Enfermeras de la Maternidad de São Paulo y el Curso de Enfermeras del Hospital São Joaquim, ambos en 1908; la Escuela de Enfermeras de la Cruz Roja Brasileña, Filial São Paulo, en 1914 (todos en la ciudad de São Paulo); y, en 1918, el Curso de Enfermeras de Pró-Matre y el Curso de Enfermeras del Policlínico Botafogo, ambos en Rio de Janeiro^(7)^.

El modelo angloamericano de enfermería Nightingale surgió a principios de la década de 1920, con la creación de la Escuela de Enfermeras del Departamento Nacional de Salud Pública, en 1922^(8)^, y contribuyó al aumento de la visibilidad de la profesión en los medios de comunicación. Se decía que los atributos que se esperaba que representasen a la enfermera eran los de los médicos en particular, e incluían la paciencia, el desinterés y la sensibilidad, que en aquella época eran especialmente distintivos de las enfermeras^(6)^. Por otro lado, Ethel Parsons lideró el proyecto de cooperación entre los gobiernos brasileño y estadounidense y transformó el desarrollo de la enfermería brasileña con identidad propia^(8)^.

Las representaciones institucionales de tres de estas escuelas - Escola Profissional de Enfermeiros e Enfermeiras, Escola Prática de Enfermeiras da Cruz Vermelha Brasileira y Escola de Enfermeiras do Departamento Nacional de Saúde Pública - tuvieron efecto en la publicidad de medicamentos entre 1920 y 1925, con 90 anuncios aparecidos en la Revista Fon-Fon^(9)^. Además, cuando se amplió el marco temporal (1910–1931), las representaciones, que también se referían a los signos de las enfermeras de las instituciones de enseñanza, mostraron que no sólo eran mensajeras de las identidades de las instituciones de enseñanza, sino también referencias representativas en las piezas publicitarias^(10)^.

La comprensión de esas representaciones históricas en el estado de Bahía, específicamente, se remonta al período de 1923 a 1925, cuando se hicieron frecuentes las campañas sanitarias y se subvencionaron las inversiones en salud pública. En la década de 1930, las Delegaciones Federales de Salud favorecieron los intercambios con la Secretaría de Salud Pública de Bahía en el campo de la educación sanitaria^(11)^. Esto se debió a la precariedad de los servicios de salud pública ofrecidos por los hospitales de Bahía^(12)^.

A partir de este escenario, el entonces rector Edgard Santos, de la Universidad Federal de Bahía (UFBA), propuso la creación de un complejo hospitalario, que requeriría el trabajo de enfermeros graduados. Para atender esa necesidad, la directora de la Escuela de Enfermería de la Universidad de São Paulo (USP), enfermera Edith de Magalhães Fraenkel, fue invitada a crear la Escuela de Enfermería de Bahía (EEB)^(11)^.

Cabe destacar que la EEB fue creada por el Decreto Ley nº 8.779, del 22 de enero de 1946, e incorporada a la UFBA, con el objetivo de formar enfermeras para prestar asistencia en los nuevos hospitales universitarios que se estaban inaugurando y, con ello, promover la educación sanitaria^(12)^.

Así, a raíz de estos acontecimientos y reconociendo la trayectoria histórica y social de la construcción de la imagen de la enfermera en la sociedad, este estudio tiene como objetivo analizar las representaciones de las enfermeras a través de la prensa escrita en Bahía, como estrategia de visibilidad de la sociedad bahiana, de 1936 a 1956.

## MÉTODO

### Tipo de Estudio

Se trata de un estudio histórico con enfoque seriado, en la dimensión de la Historia Cultural y en el campo de la historia de la enfermería y de la mujer, lo que significa, respectivamente, la tipología de la fuente secuenciada (periódico), cuando es posible investigar parte de la realidad vivida^(13)^, y, en este caso, la representación de la enfermera.

### Localización

El marco temporal de 1936 a 1956 abarca el período de 20 años durante el cual las Delegaciones Federales de Salud y la Secretaría de Salud Pública (Bahía) estuvieron involucradas en actividades de enseñanza en el campo de la salud. La trayectoria muestra que la imagen de la enfermera fue utilizada por intereses sociales, económicos y políticos a lo largo del tiempo a través de los sectores implicados. La época de (Getulio) Vargas, la Segunda Guerra Mundial y la creación de la Escuela de Enfermería de la Universidad Federal de Bahía se contextualizaron en este marco temporal, influyendo así en la formación de su identidad y visibilidad profesional.

Por lo tanto, la delimitación geográfica se limita al estado de Bahía y su principal fuente documental es el texto periodístico, concretamente la serie del periódico «A Tarde», debido a su circulación en Bahía, con una edición desde octubre de 1912 hasta la actualidad. Este periódico fue fundado por Ernesto Simões Filho (1886–1957), licenciado en Derecho y miembro fundador de la Academia Bahiana de Letras^(14)^.

### Criterios de Selección

Los criterios de inclusión utilizados fueron la presencia del término enfermera exclusivamente en femenino y la exclusión del término en masculino, así como noticias de enfermería de fuera del estado de Bahía.

### Recolección de Datos

La búsqueda de periódicos «A Tarde» se realizó en el sitio web de la Fundación Pedro Calmon, ubicada en Salvador (BA), de julio de 2018 a enero de 2019. Los hallazgos se organizaron en una hoja de cálculo *de Microsoft Excel* compuesta por las siguientes variables: fecha, título y página.

### Análisis y Procesamiento de los Datos

Se utilizó como método de análisis el proceso de triangulación de las fuentes de datos, permitiendo la síntesis y el análisis del contenido y sus posibles interrelaciones y distanciamientos de los temas^(15)^, así como la posterior discusión sobre los temas de la Historia de la Enfermería en Río de Janeiro y Bahía, articulados con la literatura de adherencia.

Las publicaciones de la base de datos se analizaron con el software ATLAS.ti (*Qualitative Research and Solutions*) mediante la codificación de los fragmentos periodísticos del periódico «A Tarde» con los términos enfermera(s) de cada reportaje. A través de este procedimiento, se aplicó la técnica de triangulación de datos, basada en criterios específicos a la luz del contexto de la noticia y su codificación para la narrativa de datos cualitativos, garantizando credibilidad y fiabilidad. Además, el enunciado descriptivo fue producido por la(s) enfermera(s) adjetivada(s), dando origen a las categorías^(16)^.

### Aspectos Éticos

La investigación siguió las normas definidas en las Resoluciones 466/2012 y 510/2016 del Consejo Nacional de Salud, así como la Ley 12.527/2011 sobre derechos de autor. El envío al Comité de Ética fue dispensado, por tratarse de un estudio documental que utilizó solamente documentos públicos de libre acceso a la población.

## RESULTADOS

En esta carrera por representar a la enfermera, se adoptaron diversos adjetivos para representar lo que esta enfermera significaba en su singularidad. Algunos de estos adjetivos fueron: Enfermera Graduada; Enfermera Práctica; Enfermera Voluntaria y Profesional; Enfermera Visitadora Social; Enfermera de Salud Pública; Enfermera Católica; Enfermera Auxiliar; Enfermera de Primeros Auxilios y Enfermera Samaritana^(17)^.

En la lucha simbólica por enunciar la representación de la enfermera, estos adjetivos fueron publicados en la prensa durante décadas, hasta la promulgación de la Ley 775/1949, que regía la enseñanza de la enfermería en el país. Esta disposición legal estableció dos denominaciones para la formación en enfermería: Enfermera y Auxiliar de Enfermería, diferenciadas por los prerrequisitos de tiempo de formación y contenidos impartidos.

Dicho esto, en la Figura 1 se detallan 542 publicaciones en el periódico «A Tarde» entre 1936 y 1956. Del análisis de estas publicaciones se desprenden tres de las representaciones más llamativas de las enfermeras de la época: *enfermera en salud pública, enfermera militar y enfermera religiosa.* Cabe destacar que, en los años 1941, 1946, 1947, 1948 y 1954 no se encontró ninguna mención al término enfermera(s).

**Figura 1:** Movimiento de publicaciones con el término enfermera(s) en el periódico A Tarde (1936–1956) - Salvador, BA, Brasil, 2019.

En este contexto, las representaciones de las enfermeras, a través del discurso serializado del periódico «A Tarde», revelan culturalmente una de las partes del dominio de la historia de las mujeres. Por lo tanto, fluctuó en tres adjetivos como una imagen mental de lo femenino en Bahía.

En la investigación en el periódico «A Tarde», los pasajes analizados con ocurrencias del término *enfermera de salud pública* se utilizaron en referencia a las graduadas de la Escuela de Enfermería del Departamento Nacional de Salud Pública / Escuela de Enfermeras Dona Anna Nery. La enfermera de salud pública en el país contribuyó a la deconstrucción de los determinantes contextuales en relación con el trabajo de las enfermeras en el país. La inserción de las enfermeras graduadas en DNSP en la sociedad trajo reconocimiento y minimizó la resistencia médica a los cuidados que abogaban por la prevención y la promoción de la salud.

A continuación, pasamos a la segunda representación encontrada en este estudio, la de la *enfermería militar.* A finales de los años 30 y principios de los 40 estalló la Segunda Guerra Mundial (1939–1945). En la época, la Cruz Roja Brasileña - órgano central en Río de Janeiro - promovió el Curso de Enfermeras Samaritanas, con énfasis en la formación de enfermeras de guerra, de acuerdo con sus principios institucionales, que se resumen en actuar en la paz y en la guerra en nombre de la humanidad^(18)^. Con este objetivo, se publicaron artículos periodísticos para atraer candidatos a la formación antes de partir para la guerra.

Así, la formación militar de las enfermeras para el frente de guerra, el viaje al extranjero y el regreso a Brasil ha sido objeto de estudios^(19,20)^. Los artículos publicados en el periódico «A Tarde» presentaban los criterios de admisión en la Fuerza Expedicionaria Brasileña: diploma de enfermera o samaritana, estado civil de soltera o viuda, edad entre 20 y 40 años, idoneidad moral y aptitud física. También vincularon esta decisión a sentimientos de patriotismo y heroísmo, asociados a la imagen de Anna Nery. Sin embargo, si nos fijamos en la representación de la enfermera militar, en el escenario bahiano había discursos que llamaban a las mujeres a ser «convocadas».

Los artículos también informaban sobre el papel de la mujer en la guerra y traían consigo la representación de la mujer heroína. Esto se refería a los signos interpretativos de dos maneras: 1) para minimizar las conciencias, cuando el símbolo está determinado por lo que se conoce; y 2) para determinar las actitudes humanas hacia él, lo que resulta en la perturbación o el mantenimiento del poder^(21)^. En este caso, ambas se transmitieron en asociación con las mujeres militares, es decir, ejemplos morales, luchadoras, de mentalidad fuerte, ejecutoras de magníficos trabajos y valientes.

En cuanto a la tercera representación encontrada, sobre la *enfermera religiosa* en el periódico «A Tarde», los artículos se asociaban a los atributos de caridad, don vocacional y benevolencia. En este sentido, relacionaban la profesión con la práctica asistencial de las religiosas (Hermanas de la Caridad) en el siglo XIX, antes del inicio de la profesionalización de la enfermería en Brasil.

Cabe destacar que el acto de caridad ejercido por las religiosas era incluso elogiado estratégicamente por los gestores hospitalarios. Esta percepción era evidente en algunos fragmentos del periódico «A Tarde», que mencionaba la imposibilidad de recibir un plus de insalubridad, como derecho laboral, porque ejercían la caridad. Así, la representación de una enfermera religiosa se revestía de sentimientos de votos doctrinales a favor de lo divino.

Otros artículos del periódico «A Tarde» se referían a la personificación de la representación de la enfermera religiosa desprovista de vanidad, que reiteraba la imagen de ángeles vestidos de blanco, ayudantes de Dios en la tierra y humanitarios^(22)^. De esta forma, la asociación reforzaba la sacralización y desdibujaba aún más quién era realmente la enfermera profesional.

## DISCUSIÓN

La década de 1920 estuvo marcada por la Reforma Sanitaria liderada por Carlos Chagas y la implantación de la Enfermería Moderna. En la época, hubo una fuerte inversión en la prensa escrita e ilustrada sobre la Escuela de Enfermeras del Departamento Nacional de Salud Pública, hoy Escuela de Enfermería Anna Nery, y también sobre la Escuela Práctica de Enfermería de la Cruz Roja Brasileña - la organización central en Río de Janeiro^(6)^. Además de éstas, otras instituciones educativas favorables a la profesionalización también se disputaban la enunciación de la representación mental de la enfermera.

Estas representaciones (*enfermera de salud pública, militar y religiosa*), identificadas en este estudio, tienen, en efecto, sus raíces en el pasado. Como se ha dicho, la Escuela de Enfermería de Bahía fue creada en 1946 por iniciativa de la UFBA^(11)^, pero para una mejor comprensión de este proceso, es necesario remontarse a la década de 1920, cuando se reconfiguraron las acciones de salud pública en el estado de Bahía. Así, el foco de las políticas sanitarias fue la salud, con énfasis en la educación sanitaria y en el control de las enfermedades infecciosas y contagiosas prevalentes, en diferentes áreas geográficas de la sociedad bahiana, en el período de 1925 a 1930, inducido por la esfera federal. Sin embargo, la Revolución de 1930 provocó el declive de esa reforma sanitaria para adaptarse a las nuevas exigencias del gobierno de la época^(23)^.

En la década de 1920, la Escuela de Enfermeras de la Subsecretaría de Salud y Asistencia Pública (EESSAP) fue incluida en el texto del Código Sanitario (1925) y sugirió la creación de una institución educativa en el estado de Bahía, considerando el Servicio de Enfermeras Sanitarias. Este servicio sería entonces centralizado en el comando de las acciones de salud pública con vistas a auxiliar a las autoridades en el campo y realizar la vigilancia higiénica, así como el control y la prevención de enfermedades infecciosas y la educación sanitaria en espacios privados y educacionales de formación básica^(24)^.

Es importante mencionar que estos requisitos para la inscripción de los estudiantes entraban en conflicto con los exigidos a los estudiantes que deseaban asistir a la Escuela de Enfermería del Departamento Nacional de Salud Pública en Río de Janeiro. La enfermera de salud pública de la década de 1920 en Río de Janeiro, de hecho, demarcó su espacio en la enfermería brasileña y en el campo de la salud bajo la dirección de las enfermeras estadounidenses Clara Louise Kieninger, Lorraine Genevi y Bertha Pullen, bajo la dirección de Ethel Parsons, de 1923 a 1931. Fue también un periodo de importantes inversiones financieras para el funcionamiento institucional de la Escuela por parte de la Fundación Rockefeller^(8)^.

Sabemos que hasta 1929, el Dr. J. J. Fontenelle, en documento encontrado en el Centro de Documentación de la Escuela de Enfermería Anna Nery (EEAN - CD, mód.A, cx. 25, doc. 55, 1930), informaba de la presencia de 93 enfermeras de salud pública egresadas de la Escuela de Enfermería del Departamento Nacional de Salud Pública. Sin embargo, el 63% de ellas ya no ejercían la profesión de salud pública y las perspectivas no eran prometedoras^(25)^. Así lo confirma otro estudio, según el cual el 70% de los graduados preferían servicios menos estresantes, como la consulta privada y/o los hospitales, a las visitas a domicilio^(26)^.

Dicho esto, partiendo de las premisas antes mencionadas, podemos ver que las enfermeras buscaban otros tipos de servicios, incluidos aquellos libres de las penurias de las visitas domiciliarias y de la exposición a agentes patógenos, con el fin de ocupar otros puestos en instituciones de salud. Sin embargo, durante la época en que Ethel Parsons estuvo al frente de la enfermería brasileña, construyó y dio visibilidad a la representación de la enfermera de salud pública al (retro)alimentar el imaginario colectivo de que las mujeres podían emanciparse económicamente siguiendo esta carrera^(8)^.

Paralelamente al éxodo de enfermeras de la salud pública hacia otros espacios, cabe destacar el fin de la subvención de la Fundación Rockefeller a la escuela, la partida de Parsons para Texas, cuando se promulgó el Decreto 20.109/1931, que convirtió a la Escuela de Enfermeras Anna Nery en la escuela oficial de formación de enfermeras en Brasil. Estos hechos pueden llevar a algunos a interpretar la disposición legal como la institucionalización nacional de la enfermería.

La representación de la enfermera en salud pública, defendida por Parsons, era prometedora a favor de la emancipación de la mujer, sobre todo económica, en lo que se refiere a la elección profesional^(8)^. Ese discurso, para la década de 1920, en pleno movimiento sufragista femenino, era motivador y osado, siempre y cuando no se tuviera conciencia de la ardua actividad en el campo, considerando el desgaste físico de las visitas domiciliarias, la exposición a agentes patógenos y la remuneración, lo que tuvo el efecto de impulsar un éxodo hacia el campo hospitalario^(25,26)^. En este sentido, el discurso publicado en el periódico «A Tarde» sobre la representación de las enfermeras de la sanidad pública apunta indicios de un llamamiento a la sensibilización.

El contexto de los años veinte y siguientes sirvió de trampolín para que las mujeres tuvieran al menos el deseo de ser enfermeras y, en consecuencia, de emanciparse económicamente. El hecho es que el discurso publicado en el periódico «A Tarde» llegó de alguna manera al público femenino y puede haber influido en la creación de la Escuela de Enfermería de Bahía (EEB) en la década de 1940, lo que reforzó los argumentos para su objetivo^(11)^.

La representación de la enfermera militar fue glorificada por la idea de la heroína avalada por Eurico Gaspar Dutra, ministro de Guerra (1942–1945), en reportajes periodísticos. Estas publicaciones estaban revestidas de adjetivos relacionados con la mujer, como amor, abnegación, servilismo y disciplina^(19)^.

El peso simbólico de la representación de la enfermera militar asociada al término heroína articulado con el recuerdo de la trayectoria de la bahiana Anna Nery fue, de hecho, un atractivo emblemático para los brasileños y especialmente para los bahianos. Por lo tanto, nos damos cuenta de que se trataba de registrar la participación de las mujeres en las páginas de la Historia Nacional en el contexto de la guerra vinculada a la Fuerza Expedicionaria Brasileña, con el fin de fortalecer esta representación en el imaginario colectivo.

Considerando la presentación de la identidad de las enfermeras en el ámbito religioso, se trata de una construcción histórica de una práctica que involucraba ideales de humildad, amor al prójimo y sumisión, demarcados con el advenimiento del cristianismo, que enseñaba esos ideales a sus fieles, pero también los imponía en las prácticas de cuidado. Esto llevó a una fuerte conexión entre los ideales cristianos y el trabajo de enfermería^(27)^.

Pensar en esta línea es comprender que la representación de la enfermera en el siglo XIX, por ejemplo, no estaba tan alejada de lo que se preconizaba en las décadas de 1910 y 1920. La afirmación nos muestra que, entre 1930 y 1950, permaneció en el discurso periodístico del periódico «A Tarde». El trabajo de enfermería arrastra influencias de los orígenes de las prácticas en salud, asociando a la mujer con conceptos de religiosidad, espíritu maternal o sumisión. Esto apunta a una cierta niebla en la representación de este profesional, dada la confusión con aspectos religiosos, incluso frente a normativas, por lo que esta distorsión ha prevalecido en el imaginario colectivo como herencia de la construcción sociocultural milenaria^(22,28)^.

Teniendo en cuenta lo anterior, entendemos que, independientemente de la representación de la enfermera en los hechos históricos publicados desde la perspectiva del periodismo (enfermera de salud pública, militar o religiosa), esta profesión quedó marcada en las mentes socioculturales como una actividad femenina y por su construcción social. Esta constatación, reiterada en este estudio, sirve de disparador mental para una reflexión crítica sobre la constitución de esta representación y su visibilidad, con el fin de concienciar sobre el verdadero papel social de las enfermeras en la sanidad, la relevancia de su trabajo y la formación de su identidad profesional.

Entre las posibles limitaciones se incluyen el uso exclusivo del término enfermera(o), en femenino, para buscar otras fuentes, y la elección de un único periódico.

Como contribuciones, este estudio pretende arrojar luz sobre las representaciones de las enfermeras en los periódicos bahianos de la primera mitad del siglo XX, lo que contribuye a otros estudios que, desde una perspectiva histórica, deben realizarse para comprender los movimientos identitarios de la profesión. También podrá subsidiar otras investigaciones sobre las representaciones de las enfermeras en Bahía y en la región Nordeste de Brasil.

## CONCLUSIÓN

La representación de la enfermera divulgada en los medios impresos del periódico «A Tarde» entre 1936 y 1956 se componía de una tríada: articulación con la salud pública, enfermera militar e imaginario religioso de la enfermera. Esta representación, construida y difundida en el periódico bahiano, fue históricamente utilizada con fines sociales, económicos y políticos a lo largo del tiempo, por los diversos sectores interesados, y llegó a conformar una identidad profesional que, hasta hoy, interfiere en el reconocimiento y visibilidad de la profesión, es decir, en la forma en que es vista y reconocida por la sociedad.

El periodo histórico comprendido entre 1936 y 1956 se caracterizó por la actividad asistencial y el trabajo de las personas que realizaban estas prácticas. En este sentido, la enfermería se consolidó como una actividad social necesaria, presente en los conflictos bélicos y en el contexto de las prácticas de salud comunitaria, como se observa en este análisis de fragmentos periodísticos de la época.

Así, este estudio ha permitido reflexionar críticamente sobre la representación de las enfermeras ante los acontecimientos históricos publicados en la prensa bahiana, lo que favorece la toma de conciencia de su papel social en la salud, la relevancia de su trabajo y la formación de su identidad profesional.

Sin embargo, llegar a este punto no significa el final de la historia. Lo que tenemos hasta aquí es la punta del iceberg sobre la representación de las enfermeras en otro nivel de investigación más allá del eje Río-São Paulo, y corresponde a otros investigadores escudriñar el tema en sus propios estados.

## Data Availability

El conjunto de datos completo que respalda los hallazgos de este estudio está disponible a pedido del autor correspondiente, Victor Porfirio Ferreira Almeida Santos.
